# Willingness to Pay for Social Health Insurance and Associated Factors among Health Care Providers in Addis Ababa, Ethiopia

**DOI:** 10.1155/2020/8412957

**Published:** 2020-04-14

**Authors:** Abel Mekonne, Benyam Seifu, Chernet Hailu, Alemayehu Atomsa

**Affiliations:** ^1^Oromia Developmental Association, Ethiopia; ^2^College of Medicine and Health Sciences, Ambo University, Ethiopia; ^3^Department of Epidemiology, Faculty of Public Health, Jimma University, Ethiopia

## Abstract

Background. Cost sharing between beneficiaries and government is critical to attain universal health coverage. The government of Ethiopia introduced social health insurance to improve access to quality health services. Hence, HCP are the ultimate frontline service provider; their WTP for health insurance could influence the implementation of the scheme directly or indirectly. However, there is limited evidence on willingness to pay (WTP) for social health insurance (SHI) among health professionals. Methods. A cross-sectional study was conducted in Addis Ababa, Ethiopia, from May 1st to August 15th, 2019. A total sample of 480 health care providers was selected using a multistage sampling method. The collected data were entered into Epi Info version 7.1 and analyzed with SPSS version 23. Binary and multiple logistic regression analysis was carried out to identify the associated factor outcome variable. The association was presented in odds ratio with 95% confidence interval and significance determined at a P value less than 0.05. Result. A total of 460 health care providers responded to the questionnaire, making a 95.8% response rate. Of the respondents, only 132 (28.7%) were WTP for SHI. Higher educational status [AOR = 2.9, 95% CI (1.2-7.3)], higher monthly income [AOR = 2.2, 95% CI (1.2-4.3)], recent family illness [AOR = 2.4, 95% CI (1.4-4.4)], and a good awareness about SHI [AOR = 4.4, 95% CI (2.4-7.8)] showed significant association with WTP for SHI. The main reasons for not WTP were thinking the government should cover the cost, preferring out-pocket payment and the provided SHI scheme does not cover all the health care costs health care providers lost interest in pay for SHI. Conclusion and Recommendation. The majority of health care providers were not willing to pay for the introduced SHI scheme. The provided SHI scheme should be clear and provide special consideration for health care providers as the majority of them receives free health care service from their employer health care institution. Also, the government, health professional associations, and other concerned stakeholders should provide awareness creation programs by targeting low and middle-level health professionals in order to increase WTP for SHI among health care providers.

## 1. Background

Health care spending increased worldwide from time to time. However, in developing countries, health care spending depends on out-of-pocket payment (1, 2). According to WHO 2012 report, in low-income countries, the share of out-of-pocket payment (OOP) measured in USD ($) terms was 48% of total expenditure compared to 14% in countries with higher incomes. Pooling reduces uncertainty for both citizens and providers. By increasing and stabilizing demand and the flow of funds, pooling can increase the likelihood that patients will be able to afford services and that a higher volume of services will justify new provider investments (1, 2). Also, this additional financial protection is seen as a way of allowing more people to use needed services without incurring high OOP payments, effectively moving closer to universal coverage (1).

Health care finance is a scheme that helps to make funding available to guarantee that everyone has the right to use public health and personal health care. It also comprises the foundation of finance to health, the time when it is available, and how the capital raised is utilized (2–4). It can be national, community, or private. They can also be mandatory or voluntary. Mandatory schemes are usually national, in which there is a legal obligation for people to pay into them and are based on the principle of social solidarity (2, 5). SHI is the possible organizational opportunities for levitation and pooling funds to finance health services (1, 6, 7). Its establishment has been advocated by the WHO as a key to achieving universal coverage of health care and to ensure access to health services, particularly for the disadvantaged in less developed countries (1, 4).

In Ethiopia, OOP spending accounts for a significant proportion of health sector spending. In 2013, 90.6% of private health expenditure in Ethiopia were from out-of-pocket (8). Given the country level of development, it is likely that households who decide to use health services could easily slip into poverty. Health spending took a substantial proportion of household disposable income, and this level of spending could be prohibitive for accessing health care services (9–11). Ethiopian Demographic and Health Survey (EDHS) 2016 shows that the Health insurance coverage is extremely low; 95% of women and 94% of men are not covered by any type of health insurance (12).

In order to alleviate the low level of health care service utilization, improving access to quality health services in an “equitable,” efficient, and sustainable way, the government of Ethiopia has launched two health insurance schemes. The first one is community-based health insurance for agriculture and informal sector. And the second scheme was SHI, which is aimed for the formal sector. The SHI was established under Article 55 (1) of the Constitution of the Federal Democratic Republic of Ethiopia under Proclamation No.690/20 (11, 13). The strategy includes health insurance as a mechanism to generate an additional source of revenue to secure financial protection for its citizens (3, 14). However, the willingness to pay (WTP) for SHI in the country is uncertain (3, 9). From the formal sector, health care providers (HCP) received a fee waiver from the hospitals they have been working. But the fee waiver only applied for the service they obtain from their employer hospital only, and the service package is not uniform (9, 15). This makes HCP different from other formal sector. Hence, HCP are the ultimate frontline service provider; their WTP for health insurance could influence the implementation of the scheme directly or indirectly. Besides, it is believed that they are aware of new laws related to health and they can be role models for their clients and the general community to adopt new behaviors. But there is limited evidence about HCP WTP for SHI. Therefore, this study tries to fill this evidence gap by accessing the level of WTP for SHI and its associated factors among HCP.

## 2. Methods

A cross-sectional study was employed from May 1^st^ to August 15^th^, 2019 in Addis Ababa, the capital city of Ethiopia. The total population of the city was 2,738,248 consisting of 1,304,518 men and 1,433,730 women. A total of 45 hospitals (11 governmental, 31 private, and 3 NGO) are found in the city. According to the Addis Ababa health administration office, an estimated 30,000 HCP are engaged in clinical and other related works in Addis Ababa.

The sample size was calculated by Epi info version 7.1 considering the following parameters; P: 74.4% of WTP for SHI (16), d = margin of error is 5%, 95% CI = Za/2 = 1.96%, 10% nonresponse rate, design effect: 1.5, and the final sample size became 480. Multistage sampling was used to select study participants. First, 15 hospitals randomly selected (5 government, 9 private, and 1 NGO) from the 45 hospitals found in Addis Ababa. Second, the sample was proportionally allocated for the selected hospitals and the actual study participants were selected using the lottery method.

Data was collected using an interview questionnaire which was prepared by reviewing similar WTP studies and modified to fit the local context (8, 11, 16–22). It was pretested among 10% of the sample size of the study participants, which were not included in the actual study. The data were collected by five public health officers and supervised by two assistant professors. Respondents were asked about their maximum WTP for SHI when they first expressed their willingness to join. Subsequently, respondents were invited to choose a lottery ticket from a stack of unmarked envelopes. Each respondent was randomly assigned to one of three initial values; 3%, 4% of monthly salary, and 5% of monthly salary. A maximum of three trials were performed with each respondent if the respondent was not satisfied with the results of the earlier bids. If the answer was “yes,” the interviewer increased the bid by 1% until the respondent says “no” and vice versa. Finally, those who chose 3% and above are considered as WTP yes (16, 17, 23).

The data were entered into Epi info version 7.1 and exported to SPSS version 23 for data processing and analysis. Descriptive data were presented in frequency with percent and mean with standard deviation. Logistic regression analysis was carried out and all explanatory variables that were significantly associated with the outcome variable in the bivariate analyses (P < 0.05) were entered into multivariate logistic regression model. Crude and adjusted odds ratios with their 95% confidence interval (CI) were determined, and statistically significant association was asserted based on P value less than 0.05. Model fitting test was performed using the likelihood ratio test, and multicollinearity was checked using the variance inflation factor.

## 3. Results

### 3.1. Sociodemographic Characteristics

A total of 460 health professionals were participated in the study making a response rate of 95.8%.

The majority of the respondents were male 267 (58.0%), and the mean age of the respondents was 29.8 years with SD for 4.8 years. From the study participants, 169 (36.7%) were Orthodox Christian by religion and 187 (36.7%) were Oromo by ethnicity. Regarding the educational status, 265 (57.6%) were degree holders, the majority of them were government employees, and 122 (26.5%) were nurses. The mean monthly salary of the respondents was 6034 ± 304 Ethiopian Birr (ETB) (Table 1).

### 3.2. Health Status and Health-Related Characteristics

Of the total respondents, 88 (19.1%) have been getting sick in the past three months and 87 (18.9%) of them reported that their family members faced illness in the past three months. 86 (18.7%) of them received medical treatment at health institutions. Of the respondents who faced illness, the majority of them self-paid the cost of the medical treatment 68 (80%) (Table 2).

### 3.3. Level of WTP for SHI

**Figure 1:**
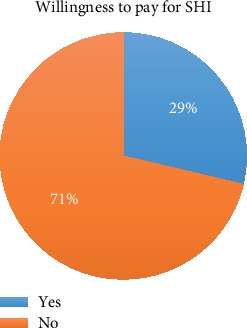
Level of WTP for SHI of health professionals in Addis Ababa, Ethiopia, April-May 2019.

The majority of the respondents had taken orientation about SRH, and 290 (63%) of them are aware of SHI. However, only 132 (28.7%) of them are willing to pay at least 3% of their monthly salary for SHI. The main reasons for not WTP were thinking the government should cover the cost, preferring out-pocket payment and the provided SHI scheme does not cover all the health care costs health care providers lost interest in pay for SHI. And few health care providers reported that the current SHI scheme is confusing, and it overlaps with the free health care service they get from their employee hospitals (Figure 1 and Table 3).

### 3.4. Factors Associated with for SHI

The independent factors associated with WTP for SHI with P value less than 0.05 were educational status, place of occupation, monthly income, history of illness in the past three months, history of illness of family member in the past three months, and awareness of SHI. In multivariate analysis, four variables found to be significantly associated with WTP for SHI with P value less than 0.05. The model fitting test was performed using the likelihood ratio test, and multicollinearity diagnosis was performed using variance inflation factor and none is detected. Study participants who had a master's degree or more were almost three times more likely to have WTP for SHI than who is a diploma holder [AOR = 2.9, 95% CI (1.2-7.3)]. Study participants whose monthly income was 4500-6500 and more than 6500 ETB are two times more likely to have a WTP for SHI [AOR = 2.2, 95% CI (1.2-4.3)] and [AOR = 2.1, 95% CI (1.2-3.6)], respectively. Study participants whose family members faced illness in the past three months were two times more likely willing to pay for SHI [AOR = 2.4, 95% CI (1.4-4.4)]. Finally, study participants who had a good awareness about SHI are four times more likely to have a WTP for SHI [AOR = 4.4, 95% CI (2.4-7.8)] (Table 4).

## 4. Discussion

This study provides important information regarding the newly launched Ethiopian SHI from HCP personal perspective. The finding of this study shows that the majority of the HCP does not have a WTP for SHI. Even though most of the studies regarding WTP for SHI are conducted at either community level or nonhealth professionals, and the prevalence of WTP for SHI in this study found to be lower than the finding of most of low and middle income Asian and Sub-Saharan African country studies. For instance, in Bangladesh, 87.6% of informal workers were willing to pay for SHI (24) and in Vietnam (72%) (25). A study conducted from Sub-Saharan African countries like Namibia shows that 87% of the study participants have WTP for SHI (16), South Sudan (68.8%) (19), southern Ethiopia (55%) (16), and in north west Ethiopia (80%) (26). The main reason for the lower prevalence of WTP for SHI among HCP in this study is that all government hospitals and most of private hospitals in Addis Ababa provide free health services for their employees and immediate family members. Because of this reason, HCP rely on the free health service rather than using SHI. But, if the health problem they face is beyond the hospital service coverage or its level, they will be forced to pay for the advanced health care they received from another hospital. However, the prevalence of WTP for SHI in this study was almost similar to a study conducted in Tanzania (30%) (27).

Regarding factors associated with WTP, educational status is found to be positively associated with WTP for SHI. This is also evidenced from studies conducted among teachers in southern Ethiopia; which explains that teachers with higher educational status are more likely WTP for SHI (13). A higher educational status also showed a positive association with WTP in different studies conducted in Iran, Bangladesh, Nigeria, and Northern Ethiopia (14, 18, 20, 21). Systematic review of WTP for health insurance in low and middle-income countries also indicated that the level of education affects WTP (7). Furthermore, the study conducted in Bangladesh concluded that “educational interventions can be used for increasing demand for health insurance scheme” (22). But on the contrary, a study from Mekele, Ethiopia, showed that the WTP decreases as the level of education increases (23). This disparity in the study from Mekele considers HCP as higher educational status among their study participants. The fact that HCP are getting health services for free, the WTP among higher educational status in this case HCP is found to be lower. Regarding family monthly income, many studies conducted in low and middle-income countries supported that better income has a positive association with WTP (7, 17, 18, 20, 23–27). For instance, a study conducted in Bangladesh revealed that WTP increased 0.196% with each 1% increase in monthly income (14). In this study, study participants whose family members faced illness in the past three months were two times more likely willing to pay for SHI. This is similar with a study conducted in Southern Ethiopia which reviled. Study participants whose family members were ill and paid for their health care service are twice more likely WTP for SHI (16). This is also found to be similar with the findings of a study conducted in Nigeria (18). Awareness about SHI is found to be associated with WTP for SHI. A study conducted in South Sudan also showed that WTP was affected by study participants' awareness of SHI (17). A study from India indicated that not only WTP is affected by awareness of SHI but awareness of SHI and WTP for SHI are also affected by similar determinant factors like gender, age, and five other sociodemographic and economic factors (24). Another study indicated that awareness about SHI not only affect WTP, but it also affects the amount to pay for SHI (15).

## 5. Conclusion and Recommendation

The majority of health care providers were not willing to pay for the introduced SHI scheme. The provided SHI scheme should be clear and provide special consideration for health care providers as the majority of them receives free health care service from their employer health care institution. Also, the government, health professional associations, and other concerned stakeholders should provide awareness creation programs by targeting low and middle-level health professionals in order to increase WTP for SHI among health care providers. This study can only be generalized for HCP providers working in primary and general hospitals found in Addis Ababa. Therefore, to address this information gap, we recommend a further study which can include all HCPs at different level of health care facility.

## Figures and Tables

**Table 1:** Sociodemographic characteristics of health professionals in Addis Ababa, Ethiopia, April-May 2019.

Variables	Response	Frequency	Percent
Sex of participant	Male	193	42.0
	Female	267	58.0

Age category	20 to 29 years old	283	61.5
	30 to 39 years old	152	33.0
	40 and older	25	5.4

Religion of participant	Orthodox Christian	169	36.7
	Muslim	129	28.0
	Protestant	156	33.9
	Other^∗^	6	1.3

Ethnicity of participant	Oromia	178	38.7
	Amhara	159	34.6
	Tigray	67	14.6
	Gurage	48	10.4
	Other^∗∗^	8	1.7

Education of participant	Diploma	165	35.9
	Degree	265	57.6
	Master's degree and above	30	6.5

Occupation	Government employee	310	67.4
	Private employee	125	27.2
	NGO employee	25	5.4

Profession	Medical doctor	63	13.7
	Nurse	122	26.5
	Health officer	97	21.7
	Laboratory technician	68	14.7
	Midwife	88	19.1
	Other^∗∗∗^	22	4.7

Monthly salary in ETB (1 USD = 29.5 ETB)	2500-4500	170	37.0
	4501-6500	154	33.5
	> = 6501	136	29.6

^∗^ Atheist and Wakefata; ^∗∗^ Wolita, Ethiopia Somali; ^∗∗∗^ Radiologist, Physiotherapist, and anesthesiologist.

**Table 2:** Health status and health-related characteristics of health professionals in Addis Ababa, Ethiopia, April-May 2019.

Variables	Response	Frequency	Percent
History of illness in the past 3 months	Yes	88	19.1
	No	372	80.9

Members who were ill	Yes	87	18.9
	No	373	81.1

Received treatment in the last 3 months	Yes	86	18.7
	No	374	81.3

Where did you get treatment?	NGO-owned health facility	13	15.1
	Government-owned health facility	44	51.2
	Private-owned health facility	29	33.7

Who covered health care cost?	Self	68	80.0
	Government	12	14.1
	NGO	5	5.9

Satisfaction of health care cost	Dissatisfied	8	9.5
	Neutral	22	26.2
	Satisfied	54	64.3

Did you borrow money to pay for health service?	Yes	13	2.8
	No	447	97.2

**Table 3:** Level of WTP for SHI of health professionals in Addis Ababa, Ethiopia, April-May 2019.

Variables	Response	Frequency	Percent
Awareness on SHI	Yes	290	63.0
	No	170	37.0

Willingness to pay	Yes	132	28.7
	No	328	71.3

Reason for not willing to pay for the scheme^∗^	Responsibility of government to cover about the scheme	47	14.3
	Out of pocket payment is better	10	3.0
	Do not need health insurance	30	9.1
	Do not cover all needy service	97	29.6
	Health insurance is confusing scheme	19	5.8
	Always in a good health	125	38.1

^∗^Multiple responses were possible.

**Table 4:** Factors associated with WTP for SHI among health professionals in Addis Ababa, Ethiopia, April-May 2019.

Variables	WTP for SHI			
	Frequency (%)		COR (95% CI)	AOR (95% CI)
	Yes	No		
Educational status				
Diploma	42 (25.5%)	123 (74.5%)	1	1
Degree	71 (26.8%)	194 (73.2%)	5.1 (2.2-11.4)^∗∗^	2.3 (0.8-6.2)
Master's degree and above	19 (63.3%)	11 (36.7%)	4.7 (2.1-10.4)^∗∗^	2.9 (1.2-7.3)^∗^
Place of occupation				
Government hospital	101 (32.6%)	209 (67.4%)	1	1
Private hospital	20 (16.0%)	105 (84.0%)	1.6 (0.7-3.7)	0.7 (0..-2.1)
NGO hospital	11 (44.0%)	14 (56.0%)	4.1 (1.6-10.3)^∗^	2.3 (0.7-6.9)
Monthly income in ETB				
2500-4500	34 (20.0%)	136 (80.0%)	1	1
4501-6500	41 (26.6%)	113 (73.4%)	2.8 (1.7-4.7)^∗∗^	2.2 (1.2-4.3)^∗^
≥6501	57 (41.9%)	79 (58.1%)	2.0 (1.2-3.2)^∗∗^	2.1 (1.2-3.6)^∗^
History of illness in the past 3 months				
Yes	43 (49.4%)	44 (50.6%)	3.0 (1.8-4.9)^∗∗^	1.5 (0.7-20.2)
No	89 (23.9%)	248 (76.1%)	1	1
Family members has been ill in the past 3 months				
Yes	43 (49.4%)	44 (50.6%)	2.7 (1.9-5.1)^∗∗^	2.4 (1.4-4.1)^∗∗^
No	89 (23.9%)	248 (76.1%)	1	1
Awareness of SHI				
Yes	115 (39.7%)	175 (60.3%)	5.9 (3.4-10.2)^∗∗^	4.4 (2.4-7.8)^∗∗^
No	17 (10.0%)	153 (90.0%)	1	1

^∗^P value less than 0.05, ^∗∗^P value less than 0.001.

## Data Availability

Full data for this research is available through the corresponding author up on request.

## Abbreviation

AOR: Adjusted odds ratio
COR: Crude odds ratio
CI: Confidence interval
EDHS: Ethiopian Demographic Health Survey
ETB: Ethiopian birr
HCP: Health care provider
NGO: Nongovernmental organization
SD: Standard deviation
SHI: Social health insurance
WTP: Willingness to pay
WHO: World Health Organization.
